# Cerebrovascular risk in rheumatoid arthritis patients: insights from carotid artery atherosclerosis in the Paracelsus 10,000 study

**DOI:** 10.1007/s00296-024-05781-4

**Published:** 2025-01-18

**Authors:** Mathias Ausserwinkler, Sophie Gensluckner, Vanessa Frey, Isabella Gostner, Bernhard Paulweber, Eugen Trinka, Patrick Langthaler, Christian Datz, Bernhard Iglseder, Jens Thiel, Hans-Joerg Neumann, Maria Flamm, Elmar Aigner, Bernhard Wernly

**Affiliations:** 1 Department of Internal Medicine, Elisabethinen Hospital Klagenfurt, Klagenfurt, Austria; 2 First Department of Medicine, Paracelsus Medical University, Salzburg, Austria; 3 Department of Neurology, Neurointensive Care and Neurorehabilitation, Member of the European Reference Network EpiCARE, Christian Doppler University Hospital, Centre for Cognitive Neuroscience, Paracelsus Medical University, Salzburg, Austria; 4 Neuroscience Institute, Paracelsus Medical University, Salzburg, Austria; 5 Department of Internal Medicine, General Hospital Oberndorf, Teaching Hospital of the Paracelsus Medical University, Salzburg, Austria; 6 Department of Geriatric Medicine, University Hospital Salzburg (SALK-Campus CDK), Paracelsus Medical University, Salzburg, Austria; 7 Division of Rheumatology and Clinical Immunology, Department of Internal Medicine, Medical University, Graz, Austria; 8 Clinic for Rheumatology and Clinical Immunology, Faculty of Medicine, University Hospital Freiburg, Freiburg, Germany; 9 Institute of General Practice, Family Medicine and Preventive Medicine, Center for Public Health and Healthcare Research, Paracelsus Medical University, Salzburg, Austria; 10 Institute of Internal Medicine, Barmherzige Brueder Hospital, Salzburg, Austria

**Keywords:** Rheumatoid arthritis, Cerebrovascular risk, Carotid artery atherosclerosis, Plaque formation, Cardiovascular risk factors, Ultrasound imaging, Intima-media thickness, Total plaque area, Systemic inflammation, Autoimmune disease, Paracelsus 10,000 study, Chronic inflammation, Metabolic syndrome, LDL cholesterol, Stroke risk

## Abstract

Rheumatoid arthritis (RA) is a chronic autoimmune disease marked by systemic inflammation. While RA primarily affects the joints, its systemic effects may lead to an increased cerebro- and cardiovascular risk. Atherosclerosis of the carotid arteries is a significant risk factor for cerebrovascular events and serves as a surrogate marker for cardiovascular risk. This study explores the link between RA and carotid artery atherosclerosis with data from the Paracelsus 10,000 Study. Baseline assessments were conducted on individuals randomly selected from Salzburg and its surrounding regions. Participants diagnosed with RA based on ACR-EULAR classification criteria and who underwent carotid artery ultrasound were included. Data were gathered from a total of 9729 participants, among whom 299 were diagnosed with RA. Carotid arteries were examined using ultrasound imaging. The primary endpoint was the difference in the prevalence of plaque presence between the RA and non-RA groups. One univariate (Model I) and three multivariate analyses were conducted, with adjustments in Model II incorporating SCORE 2, while Model III accounted for metabolic syndrome, age and sex. Additionally, Model IV included further adjustments for high-sensitivity C-reactive protein (hs-CRP). Plaque presence was defined as the ultrasound detection of plaque formation larger than 0 mm^2^, regardless of whether it was unilateral or bilateral. Additional assessments included carotid stenosis, intima-media thickness (IMT) and total plaque area (TPA). RA patients had a higher prevalence of plaque (50%) compared to non-RA individuals (38%). The odds ratio (OR) for plaque presence in RA patients versus non-RA individuals was 1.64 (95% CI 1.30–2.06). This association persisted after adjusting for SCORE2, with an adjusted odds ratio (aOR) of 1.65 (95% CI 1.26–2.15). The association remained significant when adjusting for metabolic syndrome, age and sex (aOR = 1.32, 95% CI 1.02–1.72) and also in Model IV, which included further adjustment for hs-CRP (OR = 1.33, 95% CI 1.02–1.74). The findings underscore an increased risk of cerebrovascular disease associated with RA. This study highlights the importance of thorough cerebrovascular and cardiovascular risk assessments, along with proactive management, for RA patients to reduce this risk. Recognizing the substantial impact of RA on stroke and cerebrovascular disease is important for enhancing patient care strategies. Carotid ultrasound appears to be an effective method for atherosclerosis screening in RA patients.

## Introduction

RA is the most common chronic autoimmune disease marked by systemic inflammation primally of the synovial joints. Its prevalence extends to about 1% of the global population with a notably preponderance of females [1, 2]. While RA predominantly affects joints evidence indicates its systemic impact extending beyond the musculoskeletal system to affect various organs [3]. Of particular significance is the connection between RA and cardiovascular disease (CVD) and cerebrovascular risk [4, 5]. Individuals with RA confront a substantially heightened risk of developing CVD, particularly atherosclerosis, coronary artery disease, myocardial infarction and heart failure. This also applies to cerebrovascular diseases. Stroke risk is significantly elevated in patients with various types of arthritis and rheumatic diseases, particularly in younger individuals under 50 years old, with the highest risk seen in rheumatoid arthritis and systemic lupus erythematosus. Carotid atherosclerosis and plaques are direct risk factors for cerebrovascular diseases like stroke, while serving as indirect biomarkers for cardiovascular diseases. [4, 6, 7]. This is also evident in other autoimmune disorders [5]. According to the European Society of Cardiology (ESC) guidelines, individuals with carotid plaques are at very high risk for stroke or myocardial infarction and should follow stringent low-density lipoprotein (LDL) management [8]. Several factors contribute to this elevated cardio- and cerebrovascular risk among RA patients encompassing chronic inflammation, conventional cardiovascular risk elements and potential side effects of RA medications, notably glucocorticoids [9].

Chronic inflammation, a characteristic feature of RA, is increasingly acknowledged as an essential factor in the onset and progression of atherosclerosis, so RA seems to be a suitable model for investigating the interplay between chronic non vascular systemic inflammation, cerebrovascular disease [5]. Studies have demonstrated that achieving a state of low disease activity or remission in RA patients can return the vascular risk comparable to the general population [10]. Despite the substantial evidence linking RA with an increased cerebro- and cardiovascular risk the underlying mechanisms remain only partially understood.

The reasons for the increased risk are heterogenous and not all the pathways are well known. Potential factors could be the following: The persistent inflammatory state in RA contributes to endothelial dysfunction, oxidative stress and dyslipidemia which promote atherosclerosis [11]. Endothelial dysfunction is characterized by impaired vasodilation, increased vascular permeability and a prothrombotic state and is an early event in atherosclerosis. Autoimmune mechanisms may directly contribute to the development of CVD. For example, autoantibodies targeting oxidized LDL have been implicated in the pathogenesis of atherosclerosis [12]. It is known that medications like glucocorticoids which are used to manage rheumatic diseases may exacerbate cardiovascular risk. Glucocorticoids can induce insulin resistance, hypertension, dyslipidemia and promote a prothrombotic state [13]. Joint pain and fatigue which are often associated with rheumatic diseases lead to reduced physical activity levels and this could turn to obesity and insulin resistance and so to higher risk of CVD [14]. Patients with rheumatic diseases often experience higher levels of psychological distress, including depression and anxiety, which have been independently associated with an increased risk of CVD [15]. In addition to chronic inflammation, patients with rheumatic diseases often have an increased prevalence of traditional cardiovascular risk factors such as hypertension, dyslipidemia, obesity, and smoking [16].

The Paracelsus 10.000 Study offers an opportunity to investigate the association between RA and carotid artery atherosclerosis in an Austrian population [17]. The dataset from this cohort allows us to compare the difference in the occurrence of atherosclerosis between RA-patients and the general population [18–21]. Our study aims to address a critical gap in understanding the relationship between RA and cerebrovascular risk, particularly through the lens of carotid artery atherosclerosis. While prior research has established an elevated cardiovascular risk in RA patients, the mechanisms linking RA with subclinical and cerebrovascular disease remain not that clear. By leveraging data from the Paracelsus 10,000 Study, our research uniquely investigates the prevalence and characteristics of carotid plaques in an RA population, highlighting their role as risk factor for stroke and surrogate marker for cardiovascular risk. These findings contribute to clinicians' ability to tailor risk assessments and management strategies for RA patients and provide researchers with a robust dataset for exploring the interplay between chronic inflammation and vascular health.

## Methods

**Fig. 1:**
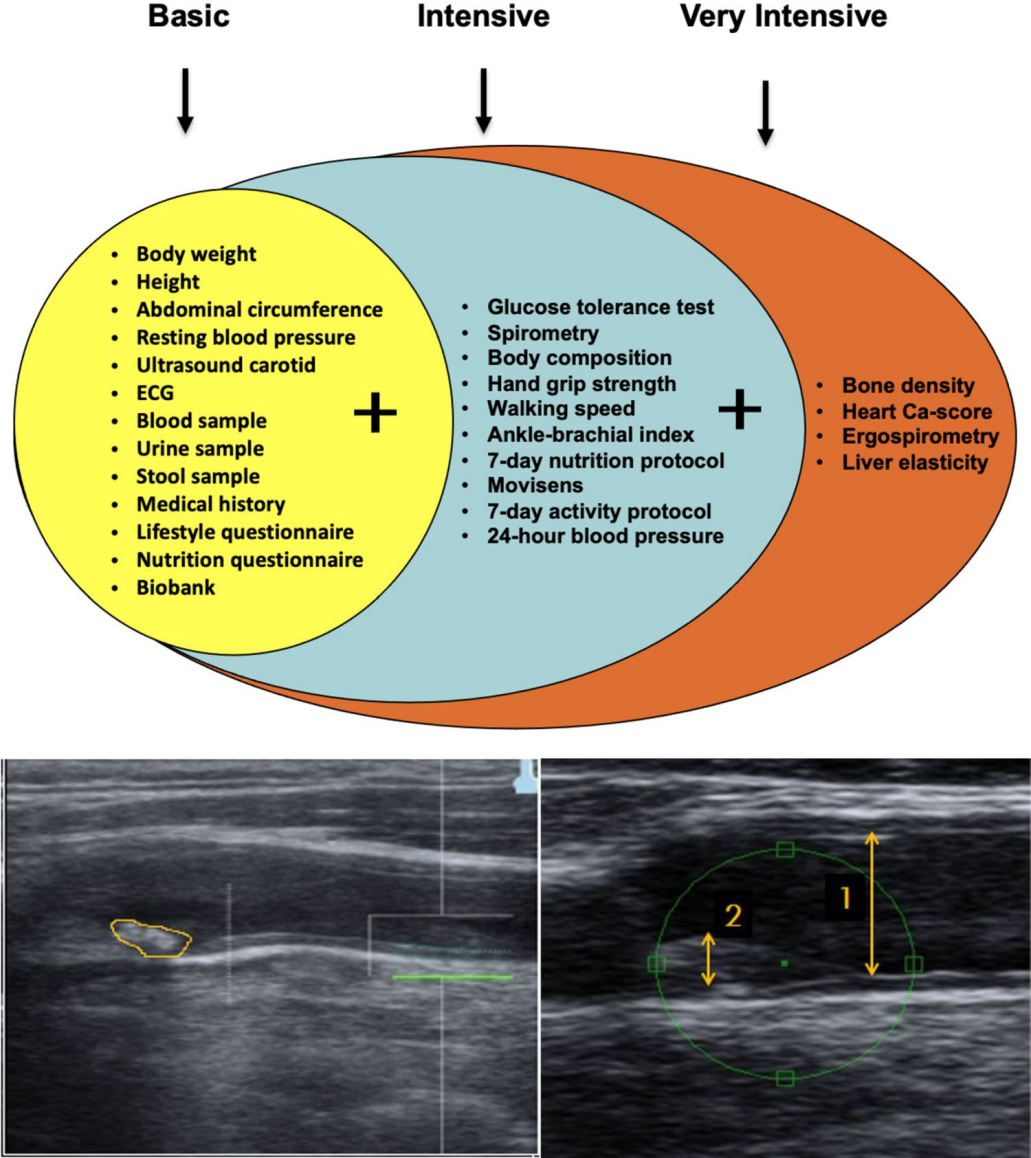
Illustrates the three levels of medical examinations. The two ultrasound images reveal a transverse view of the carotid artery with evidence of plaque deposition. The degree of stenosis in this carotid artery indicates a moderate risk level. Longitudinal section of the carotid artery, where blood appears as anechoic. Plaque deposits reflected as varying echogenicity from white to gray

This retrospective study used data from the Paracelsus 10.000 cohort, a population-based observational study conducted in the city of Salzburg and its surrounding areas. The cohort consisted of participants aged between 40 and 77 years who underwent baseline assessments between April 2013 and March 2020. Recruitment for the study aimed to randomly select individuals from the population of Salzburg based on records from the Austrian national registry of residents. Approximately 56,600 invitation letters were sent out, resulting in a total of 10,044 participants being examined. All participants underwent a standardized series of clinical, laboratory and imaging assessments, ensuring consistency across the cohort. The assessments included Body Mass Index (BMI), waist and hip circumference measured with a flexible tape for assessing central obesity, body composition analysis performed in a subset of participants using bioelectrical impedance analysis. Blood samples were collected after an overnight fast. Key biomarkers analyzed included lipid profiles [total cholesterol, LDL, high-density lipoprotein (HDL) and triglycerides]. Glucose metabolism markers (fasting glucose and hemoglobin A1c HbA1c), inflammatory markers like hs-CRP and renal and liver function. Cardiovascular Assessments were done with blood pressure measured bilaterally in a seated position, repeated three times per side after a 60-s resting interval, electrocardiography (ECG). Ankle-Brachial Index (ABI) measured three times in a supine position, 24-h ambulatory blood pressure monitoring (participants wore a portable device recording blood pressure and pulse every 15 min during the day and every 30 min at night) and ultrasound examination of the carotid arteries. In clinical examinations participants completed structured interviews to capture personal medical history, medication use and lifestyle factors (smoking, alcohol use, physical activity) and psychological assessments, including questionnaires on mental health and stress. The study primarily focused on collecting data related to medications addressing cardiovascular and metabolic risk factors, including antihypertensives, diabetes treatments and statins. Specific treatments for rheumatoid arthritis, such as antirheumatic drugs, were not a primary focus of the data collection process [17]. See Fig. 1

We included those 9729 participants of whom we had the information if RA was present or not as well as carotid plaque measurements were performed in the study. Individuals diagnosed with RA were identified based on ACR-EULAR classification criteria. This is a standardized system used to diagnose RA. A total score of 6 or more out of 10 is required for a definitive RA diagnosis. The four key domains are joint Involvement, serology (rheumatoid factor (RF) and anti-cyclic citrullinated peptide (anti-CCP) antibodies), acute-phase reactants and symptom duration [22]. The control group comprised individuals without RA from the Paracelsus 10,000 cohort, in this group patients with a diagnosis of chronic inflammatory bowel diseases and other rheumatic diseases were excluded. More than 90% of the ultrasound examinations were conducted by a single, highly experienced operator. All scans were performed on both carotid arteries while the patient was in a supine position, utilizing the same Panasonic GM–72P00A machine (Panasonic Healthcare Diagnostics US). Plaques were identified as deposits on the vessel wall with a diameter exceeding 1.5 mm and an area greater than 2.9 mm^2^. Multiple measurements of each plaque were taken from different transducer positions to enhance accuracy. Plaque morphology was classified using the Gray-Weale scoring system (types 1–4) [23] Table 1. Stenosis was noted when the vessel lumen was reduced by more than 20–30%, following ECST guidelines. Total plaque area was calculated as the sum of all plaque surfaces in the common carotid artery, internal carotid artery (including the bulb and proximal segment), and external carotid artery on each side (left and right). All imaging data were archived in the hospital's imaging system for future reference, and results were also logged in the Paracelsus 10,000 database.[17, 24, 25]. IMT measures the thickness of the inner layers of an artery and is predominantly an indicator of age [26]. TPA quantifies the extent of atherosclerotic plaque buildup within arterial walls, indicating cardiovascular disease risk [27, 28]. See Image 2 + 3.

**Table 1:** Describes the Gray-Weale classification

Gray-Weale classification	Description
Type1	Echolucent
Type2	Predominantly echolucent
Type3	Predominantly echogenic
Type 4	Echogenic

### Statistics

We analyzed continuous parameters using median and inter-quartile range, and calculated *p*-values using the Wilcoxon rank-sum test. Categorical data were expressed as percentages and compared using chi-squared tests, with a two-sided significance level of *p* < 0.05. Additionally, the study incorporated exploratory statistical analyses, including logistic (for the binary endpoints) and linear (for the continuous endpoints) regression models. These statistical models were calculated for descriptive purposes.

The primary exposure was the diagnosis of RA. The primary endpoint was the presence of any plaque (binary variable), secondary endpoints were the total plaque area (continuous variable), the presence of any stenosis (binary variable), the intima media thickness (continuous variable). We fitted three regression models: an univariate model consisting of only the dependent variable and the primary exposure and two multivariable models: one model adjusted for SCORE2 (Model II), one model for metabolic syndrome, age and sex (Model III) and one model was Model III + hsCRP (Model IV). We used Stata 18/BE for all analyses. The ESC Score2 is a risk prediction model developed by the ESC to estimate the 10-year risk of cardiovascular events in individuals. It includes traditional risk factors such as age, sex, smoking status, blood pressure, cholesterol levels and the presence of diabetes to provide a personalized risk assessment and LDL target values can be defined [29].

## Results

**Fig. 2:**
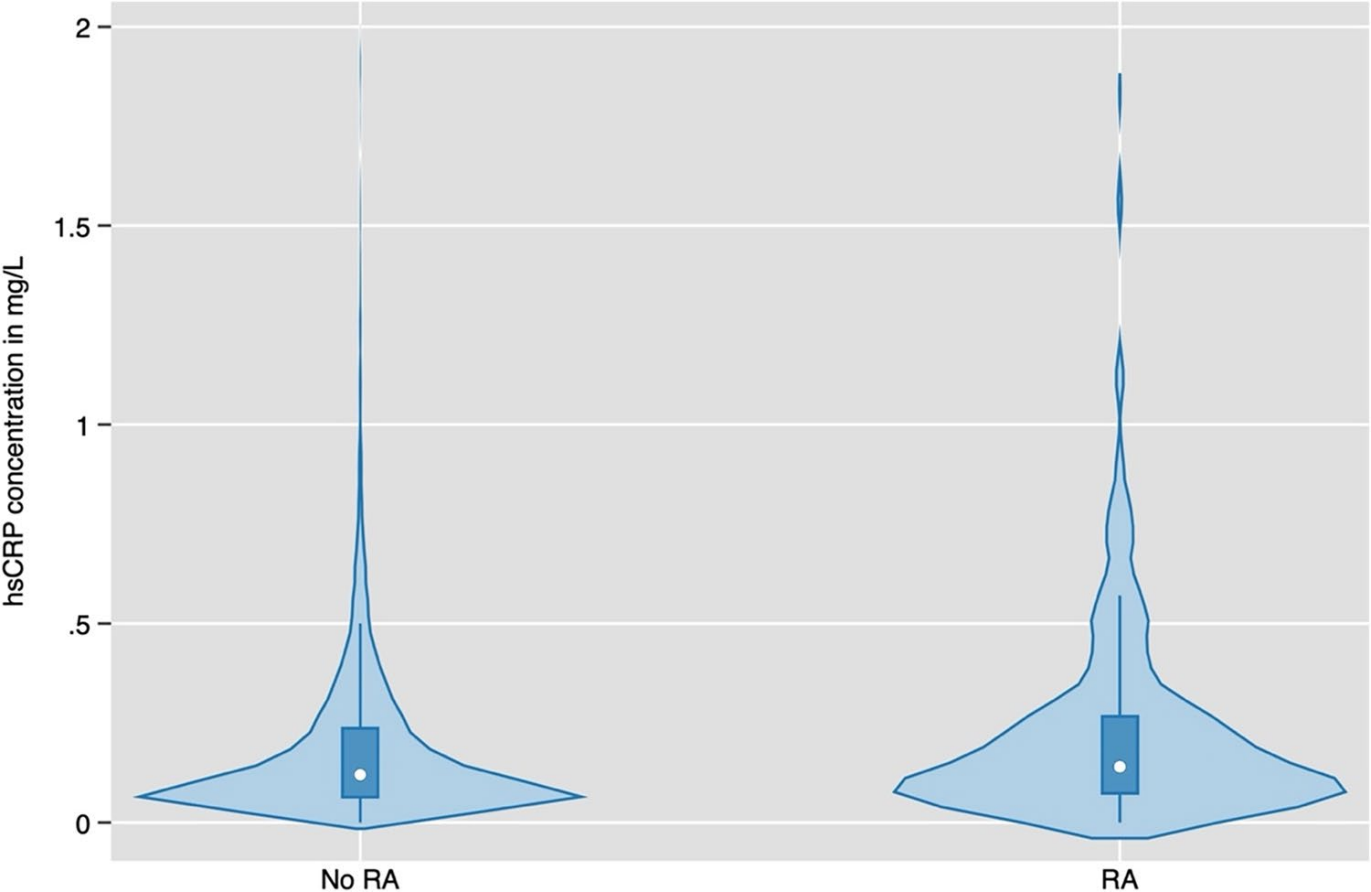
The graphic shows the distribution of hsCRP in mg/L using vioplot command [43] in patients without and with RA

Data from 9729 participants were collected, with 299 of them diagnosed with RA, 9430 were non-RA individuals. Baseline characteristics are presented in Table 2. RA patients had a slightly higher median age of 59 years compared to non-RA individuals (55 years). The population consisted of 48% males and 52% females. Among RA patients there was a higher percentage of females (69%) compared to males (31%). The hsCRP-levels were significantly higher in the RA group (median 0.14 mg/dl, 0.07–0.28) compared to the non-RA group (median 0.12 mg/dl, 0.06–0.24, p = 0.002). The graphical representation of the hsCRP-difference can be seen in Fig. 2*.*

**Table 2:** Compares various demographic and clinical characteristics between RA patients within the overall study population

	Non RA population	RA patients	p-value
	N = 9430	N = 299	
Age	55 (50–61)	59 (54–64)	< 0.001
*Age categories*			< 0.001
Age 40–49	25% (2329)	9% (28)	
Age 50–59	43% (4053)	44% (132)	
Age 60–69	28% (2638)	39% (117)	
Male	49% (4608)	31% (92)	< 0.001
Female	51% (4822)	69% (207)	< 0.001
hs-CRP md/dl	0.12 (0.06–0.24)	0.14 (0.07–0.28)	0.002
CRP mg/dl	0.12 (0.06–0.27)	0.16 (0.08–0.28)	< 0.001
Creatinin mg/dl	0.9 (0.7–1.0)	0.8 (0.7–0.9)	< 0.001
ALT U/l	22 (17–30)	22 (17–31)	0.51
AST U/l	23 (19–27)	23 (20–28)	0.47
HbAIc %	5.4 (5.3–5.6)	5.5 (5.3–5.7)	0.009
GammaGT U/l	22 (15–35)	22 (14–35)	0.71
LDL-cholesterol mg/dl	140 (116–165)	133 (114–157)	0.008
Type ll diabetes	4% (359)	6% (18)	0.051
Hypertonia	22% (2102)	35% (106)	< 0.001
Diagnosed CAD	2% (188)	6% (17)	< 0.001
*BMI categories*			< 0.001
BMI < 18.5	1% (99)	1% (3)	
BMI 19.5–24.9	40% (3807)	33% (99)	
BMI 25–29.9	39% (3708)	32% (97)	
BMI 30–34.9	14% (1354)	23% (68)	
BMI 35–39.9	4% (331)	9% (27)	
BMI > 40	1% (121)	2% (5)	
Metabolic syndrome	17% (1551)	26% (78)	< 0.001
Alcohol (g/day)	7 (2–18)	7 (2–16)	0.26
*Smoking categories*			0.074
Never smoker	45% (4022)	39% (111)	
Previous smoking	36% (3241)	43% (121)	
Current smoker	18% (1643)	18% (51)

The median levels of creatinine and liver enzyme levels were similar between RA patients and non-RA individuals. RA patients have a slightly lower median LDL-cholesterol level compared to non-RA individuals. The prevalence of Type 2 Diabetes Mellitus appeared slightly higher among RA patients, but the difference was not statistically significant (p = 0.051). RA patients had a significantly higher prevalence of hypertension compared to non-RA individuals and had a significantly higher prevalence of coronary artery disease (CAD) and metabolic syndrome compared to non-RA individuals. RA patients also had higher percentages in the overweight and obese categories. Median alcohol consumption per day was similar between RA patients and non-RA individuals. RA patients had a higher percentage of previous smokers compared to non-RA individuals.

50% of individuals with RA and 38% of those without RA exhibited any level of plaque deposition (Fig. 2). The OR for plaque presence in RA patients versus non-RA individuals was 1.64 (95% CI 1.30–2.06). This association persisted after adjusting for SCORE2, with an aOR of 1.65 (95% CI 1.26–2.15). Furthermore, when controlling for metabolic syndrome, age and sex, the association remained significant (aOR = 1.32, 95% CI 1.02–1.72) and also in Model IV, which included further adjustment for hs-CRP (OR = 1.33, 95% CI 1.02–1.74).

The inter-quartile ranges of TPA were 0.00–11.20 mm^2^ compared to 0.00–18.15 mm^2^ in the RA-population. The assessment of TPA using linear regression demonstrated that individuals with RA exhibited a TPA 5.5mm^2^ higher than those without RA (p < 0.001) with a 95% confidence interval ranging from 2.83 to 8.22. Upon adjusting for Score2, the disparity in plaque area decreased to 3.44 mm^2^ but remained statistically significant (95% CI = 1.15 to 5.72, p = 0.003). In Model III the difference in plaque area was 2.93 mm^2^ (95% CI = 0.49 to 5.37, p = 0.019). Regarding the difference in IMT between RA patients and non-RA participants, it was found to be 0.037mm in Model I (95% CI = 0.021–0.053) in Model II 0.027mm (95% CI = 0.013–0.041) and 0.013mm in Model III (95% CI = − 0.0001–0.026). The results concerning the difference in the presence of any stenosis (ECST > 0%) revealed that 33% of RA patients had stenosis compared to 26% of the non-RA group (OR = 1.42, CI = 1.11–1.80). The endpoints are summarized in Table 3.

**Table 3:** Compares the presence of the primary and secondary endpoints (plaques and severity of stenosis) between RA patients and the non-RA population

	Non RA population	RA patients	p-value
Any plaque	38% (3584)	50% (149)	< 0.001
ECST > 0%	26% (2417)	33% (99)	0.003
*Cerebrovascular stenosis categories*			0.027
No stenosis	74% (7002)	67% (199)	
ECST < 50%	25% (2369)	32% (96)	
ECST 50–69%	0% (37)	1% (2)	
ECST 70–79%	0% (7)	0% (1)	
ECST > 80%	0% (4)	0% (0)	
CIMT (mm)	0.67 (0.58–0.76)	0.70 (0.62–0.80)	< 0.001
Plaque area (mm^2^)	0.00 (0.00–11.20)	0.00 (0.00–18.15)	< 0.001

## Discussion

The results of our study underscore a significant association between RA and increased cerebrovascular risk, as evidenced by a higher prevalence of carotid artery plaque in RA patients compared to the general population- This strong association persisted even after adjusting for key confounding factors, including metabolic syndrome, age and sex. Furthermore, RA patients exhibited a significantly larger TPA and a higher likelihood of carotid artery stenosis, emphasizing the impact of RA on subclinical atherosclerosis. These findings provide robust evidence that chronic inflammation associated with RA accelerates the development of atherosclerosis, increasing the risk of cerebrovascular events such as stroke. Importantly, carotid ultrasound emerged as an effective and practical tool for detecting early signs of atherosclerosis in RA patients, offering a valuable method for risk stratification and early intervention. Chronic inflammation plays an essential role in the onset and progression of atherosclerosis, making RA an ideal model for investigating the complex interplay between chronic inflammatory diseases and cardio-and cerebrovascular diseases [5, 6, 30]. The Paracelsus 10,000 Study provides a unique opportunity to explore the association between RA and carotid artery atherosclerosis [17]. Carotid ultrasound is an efficient, non-invasive, and cost-effective method for detecting subclinical atherosclerosis in patients with RA. This technique aids in identifying carotid plaques, which, according to ESC guidelines, classify RA patients as being at very high risk for cardiovascular events, necessitating strict LDL-cholesterol management. By enabling early detection of atherosclerosis, carotid ultrasound facilitates timely intervention and individualized cardiovascular risk management. Its simplicity and accessibility make it an essential tool in the comprehensive care of RA patients. [31, 32]. Unlike the clear recommendations for screening and managing atherosclerosis in diabetes patients, guidelines for rheumatoid arthritis (RA) patients remain less well-defined, despite their similarly elevated cardiovascular risk [33]. Achieving low disease activity or remission in patients with rheumatoid arthritis should be a primary objective for rheumatologists, as it not only improves overall disease outcomes but also significantly reduces vascular risk, aligning it with that of the general population [10] (Table 4).

**Table 4:** Shows the likelihood of plaque presence in RA patients compared to non-RA individuals, in the three models

	Description	OR	CI	p
Model I	Unadjusted	1.64	1.30–2.06	< 0.001
Model II	Adjusted for Score 2	1.65	1.26–2.15	< 0.005
Model III	Adjusted for Age, Sex and Metabolic Syndrome	1.32	1.02–1.72	0.036
Model IV	Adjusted for Age, Sex, Metabolic Syndrome and hsCRP	1.33	1.02–1.74	0.046

The primary findings of this study illuminate a robust correlation between RA and carotid artery stenosis, a relationship that persists even after adjustments for potential confounders including SCORE 2, metabolic syndrome, age, sex and hsCRP. Individuals with RA exhibit also a higher total plaque area and an elevated prevalence of any stenosis in comparison to their counterparts without RA. In our view, the study's strengths lie in several key areas. Firstly, the considerable sample size of 9729 participants facilitates robust statistical analyses and enhances the generalizability of the findings. Secondly, the population-based design supports the study's external validity, ensuring that the results were applicable beyond the immediate study population. Additionally, the comprehensive assessment of the association between RA and carotid artery atherosclerosis, encompassing various outcomes such as plaque presence, total plaque area, intima media thickness and stenosis, offers a detailed understanding of the relationship between RA and cerebrovascular risk. Moreover, the adjusted analyses for potential confounders like age, sex, metabolic syndrome and SCORE 2 helps to reduce the impact of extraneous variables on the observed associations. However, the study also presented certain limitations that deserve consideration, like its retrospective design. In our study, the proportion of patients diagnosed with RA was higher (3%) than the expected prevalence. This suggests a recruitment bias, as RA patients often feel medically underserved or underdiagnosed, making them more likely to participate in studies. Similar observations have been noted in other studies [34–36]. The CRP differences, though statistically significant, were marginal, this suggests the need to look more closely at other inflammatory and immunological markers. On the other hand, it is promising that the life expectancy of RA patients has recently come much closer to that of the general population. This progress is attributed to enhanced treatments and interdisciplinary patient care. RA management should not only to control disease activity it is also important to identify and address traditional cardiovascular risk factors [37–39] (Table 5).

**Table 5:** Compares Score2, age, gender, diabetes, HbA1c, hyperlipidemia, cholesterol, LDL, HDL, triglycerides, BMI, smoking status and hypertension) between the non-RA group and RA group

	Non-RA group	RA group
SCORE2 10-year CVD risk (%) (median)	4.0 (2.1–6.8)	4.8 (2.6–7.9)
Age (median)	55 (50–62)	59 (54–64)
Gender (male/female)	49% / 51%	32% / 68%
Diabetes	4% (372)	7% (21)
HbA1c (median)	5.4 (5.3–5.6)	5.5 (5.3–5.7)
Hyperlipidemia	12% (1,176)	18% (55)
Cholesterol (median)	209 (184–235)	208 (185–229)
LDL (median)	140 (116–165)	133 (114–157)
HDL (median)	61 (50–74)	63 (51–77)
Triglycerides (median)	97 (71–137)	100 (74–140)
BMI (median)	26 (23–29)	27 (24–31)
Smoking status	45% never/36% former/18% current	39% never/43% former/18% current
Hypertension	50% (no)/50% (yes)	40% (no)/60% (yes)
Systolic blood pressure (median)	128 (118–139)	130 (119–142)

A key limitation of our study is the lack of detailed information on specific antirheumatic drugs, including their types, dosages and durations, which prevented us from fully assessing the potential impact of these medications on carotid plaque risk. Assessing the long-term cardiovascular effects of various RA treatments, such as conventional Disease-Modifying Antirheumatic Drugs (DMARDs), biologics, Janus Kinase (JAK)-inhibitors and glucocorticoids could help in understanding how these treatments influence cardiovascular risk apart from their effects on RA [9, 40]. The absence of disease activity scores, limits our ability to evaluate the relationship between disease activity and cerebrovascular risk. The investigation of the impact of the duration and severity of RA on the progression of carotid artery atherosclerosis could provide deeper insights. It's plausible that longer disease duration or more severe RA could correlate with greater vascular changes [10]. Addressing these limitations in future studies, by including comprehensive data on antirheumatic treatments and disease activity measures, will provide a more complete understanding of these associations.

**Fig. 3:**
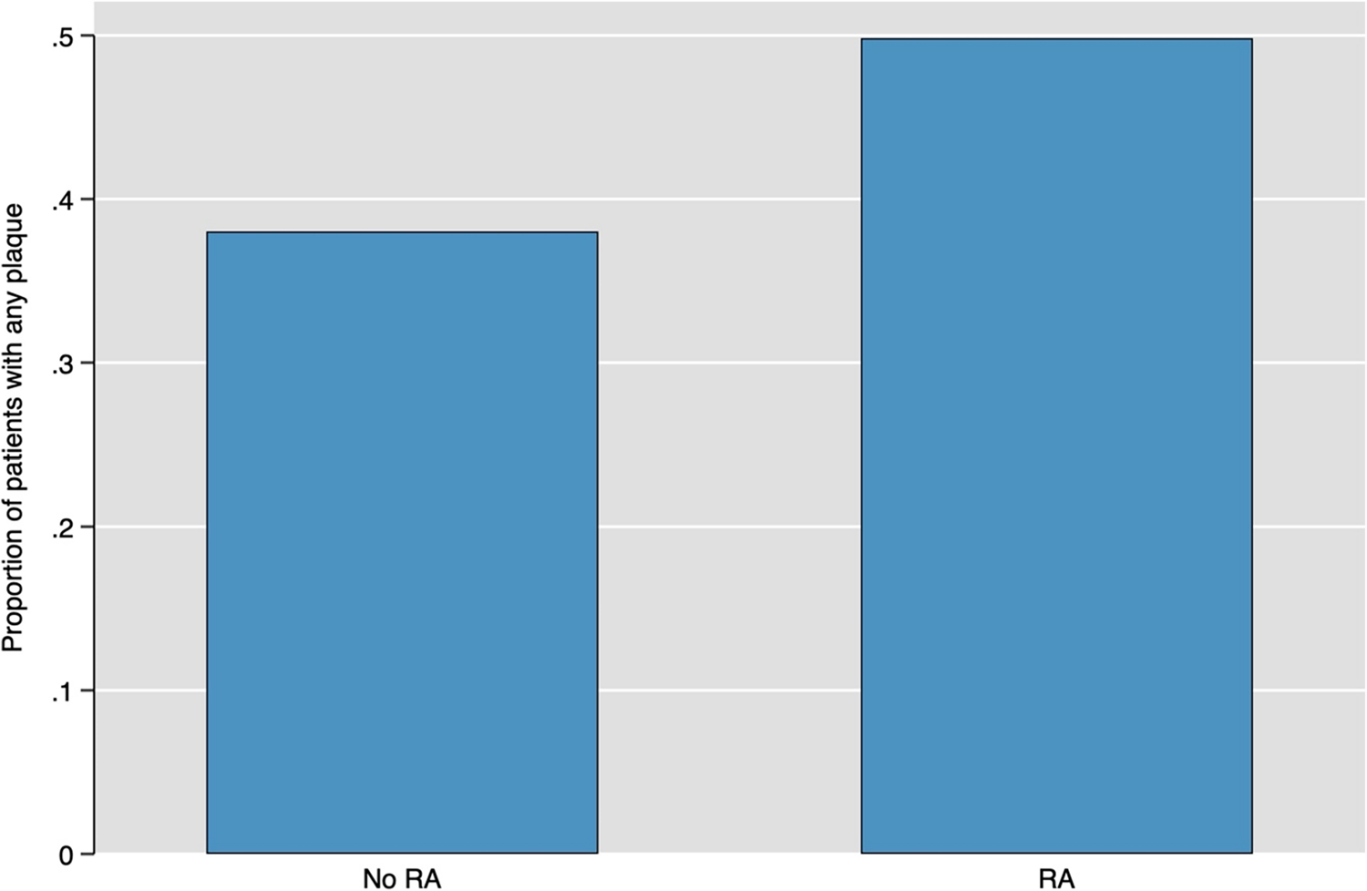
Shows the proportion of patients with any carotid plaque by rheumatoid arthritis status (38 vs. 50%)

Additionally, comparing RA with other inflammatory diseases like systemic lupus erythematosus (SLE) or psoriatic arthritis (PsA) in terms of cerebrovascular risk could clarify whether the observed vascular changes are specific to RA or common to systemic inflammatory conditions [5]. Exploring genetic markers that might influence both RA and cardiovascular risk could help identify patients at higher risk and tailor prevention strategies [41] (Fig. 3).

In summary, this research highlights the importance of recognizing RA as a significant contributor to cerebrovascular risk. It emphasizes the requirement for thorough evaluations of cardio- and cerebrovascular health and the adoption of proactive treatment approaches that address the distinct concerns and difficulties encountered by individuals with RA. Patients with rheumatic disease are especially advised to lead a healthy lifestyle, engage in regular physical activity, avoid nicotine and have their lipid levels managed by healthcare providers [42]. Remission should be the primary goal of the rheumatologic therapy, while substances augmenting vascular risk such as glucocorticoids should be avoided [9, 10].

## Data Availability

The data used in this study are available upon reasonable request. Please contact the corresponding author for further information.
